# Neurodevelopmental Outcomes at 42 Months After Thyroxine Supplementation in Infants Below 28 Weeks' Gestation: A Randomized Controlled Trial

**DOI:** 10.1089/thy.2019.0293

**Published:** 2020-07-10

**Authors:** Sze May Ng, Mark A. Turner, A. Michael Weindling

**Affiliations:** ^1^ Department of Women's and Children's Health, Institute of Translational Medicine, University of Liverpool, Liverpool, United Kingdom.; ^2^ Department of Paediatrics, Southport and Ormskirk Hospital NHS Trust, Ormskirk, United Kingdom.

**Keywords:** thyroxine supplementation, RCT, extreme preterm, neurodevelopment

## Abstract

***Background:*** Infants below 28 weeks' gestation have low thyroid hormone plasma levels compared with more mature infants and this may contribute to their risk of developmental disability. We aimed at determining the effect of supplementation with levothyroxine (LT4) for extremely premature infants born below 28 weeks' gestations on neurodevelopmental outcomes at 42 months.

***Methods:*** An explanatory double-blind, randomized, placebo-controlled trial consecutively recruited 153 infants below 28 weeks' gestation from 5 neonatal units in the United Kingdom. Infants were either supplemented with LT4 started intravenously during the first 5 days after birth and then changed to oral LT4 when enteral feeds were fully established (8 μg/kg birthweight/day as a single daily dose) or given placebo until 32 weeks' corrected gestational age. Neurodevelopmental outcomes at 42 months (range 40–43) were evaluated in 59 of these infants (30 LT4-supplemented, 29 placebo) by using Bayley III Mental and Psychomotor Developmental Indices. Cognition outcomes was correlated with plasma free thyroxine (fT4) level at 36 weeks and diffusion tensor imaging (DTI) markers.

***Results:*** The LT4 supplemented group performed significantly better in motor, language, and cognitive function domains. The mean of the difference between each group (95% confidence intervals [CI], *p*-value) was motor domain 6.96 ([0.55–13.38], *p* = 0.034); language domain 8.93 ([0.16–17.70], *p* = 0.041); and cognition domain 6.35 ([0.14–12.55], *p* = 0.045). Neurodevelopmental outcome at 42 months had some associations with the trial's primary outcome (subarachnoid space width and motor outcome, *p* = 0.03), plasma fT4 level at 36 weeks (fT4 and cognition outcome, *p* = 0.01), and DTI at 36 weeks with cognition outcomes (*p* > 0.05).

***Conclusion:*** Our data suggest that early supplementation with LT4 may improve long-term neurodevelopment in infants born below 28 weeks' gestation, but larger trials are warranted as the current reported improvements shown are not strong enough to warrant a change in practice.

## Introduction

Among infants born at term, the disability associated with hypothyroidism in the immediate postnatal period can be prevented by giving supplementary thyroid hormone: Even mild thyroid hormone insufficiency can produce measurable, but preventable deficits in neuropsychological functions (1). Infants born extremely prematurely have a high incidence of motor and cognitive deficits that are worse at lower gestations (2). Infants born at extreme prematurity (before 28 weeks gestation) are at high risk of developmental disability (3,4), and up to 69% of infants born below 30 weeks' gestation have low free thyroxine (fT4) concentrations compared with term infants (4–8). It is unclear whether the condition known as transient hypothyroxinaemia of prematurity (9) contributes to the causation of neurodisability and should be treated or whether it is a marker of illness severity or a normal concomitant of prematurity.

Some steps have been taken to address this problem. A phase 1 trial of differing doses and modes of administration of thyroid hormones to 168 preterm neonates less than 28 weeks' gestation found no difference in neurodevelopmental outcomes in relation to thyroid hormone treatment at 36 months of age (10). A previous large trial of levothyroxine (LT4) supplementation recruited 200 infants <30 weeks' gestation and found no differences in outcomes for the whole sample, but a subgroup of 46 infants born <28 weeks' gestation showed an improved Bayley Mental Development Index (MDI) and Psychomotor Development Index (PDI) at 2 years of age (7,11). In the same study, survivors of the trial were assessed at the age of 5.7 years and an improved IQ score (effect size 0.65 of standard deviation [SD]) was found in favor of the most immature infants <27 weeks' gestation from the LT4-supplemented group (12). Another study found that early replacement therapy with LT4 in doses of 5–10 μg/kg/day initiated in the second week of life was associated with improved long-term mental development at 7 years of age in premature infants born with low birth weight <2500 g (13).

Details and early results of the TIPIT trial have been previously reported (14,15). In brief, TIPIT explored the effect of early supplementation with LT4 (8 μg/kg birthweight/day as a single daily dose) started intravenously during the first 5 days after birth and then changed to an oral preparation when enteral feeds were fully established and given until 32 weeks' corrected gestational age (CGA). There were no differences at 36 weeks' CGA between infants given LT4 (*n* = 78) or placebo (*n* = 75) for width of the subarachnoid space, head circumference, body weight, or mortality. Although infants who received LT4 supplementation had significantly shorter leg lengths at 36 weeks' CGA (*p* = 0.02), there was no statistical difference when the analysis was adjusted for baseline length (14). TIPIT aimed at investigating whether early T4 supplementation improves brain growth measured at term equivalence, using brain size as a surrogate outcome measure for neurological development. Multiple factors are likely to be implicated in normal brain development, and the TIPIT study focused on the status of thyroid axis at a time that neuronal migration has just been completed and synaptogenesis is taking place. Thyroid hormones are known to improve brain connectivity; therefore, improving central nervous connectivity at this vulnerable stage of development might reasonably be expected to improve higher functioning. Diffusion tensor imaging (DTI) makes it possible to observe white matter pathways before myelination is evident on conventional magnetic resonance imaging (MRI) and it enables measurements of apparent diffusion coefficient (ADC) and fractional anisotropy (FA), which are sensitive to the integrity and organization of the white matter microstructure (16).

The study reported here was a planned extension to the TIPIT trial and aimed at exploring the effects of early LT4 supplementation to extremely premature infants on their neurodevelopmental outcomes at 42 months. These outcomes were also related to selected assessments done during the initial TIPIT trial such as DTI markers to assess their value as surrogate outcomes for neurodevelopment.

## Methods

TIPIT was a multi-center, randomized, double-blind, placebo-controlled explanatory trial of postnatal LT4 supplementation given until 32 weeks' CGA to infants born below 28 weeks' gestation. Multiple births were included with both infants randomized to the same arm. The following were excluded: infants born to mothers with known thyroid disease or on antithyroid medications or amiodarone; infants with major congenital or chromosomal abnormalities known to affect thyroid function or brain development and maternal death. All infants previously recruited to the TIPIT study (14,17) were eligible to take part in this follow-up study. A nested MRI study was done on 45 infants in the TIPIT study and was previously reported (15).

A sample size of 30 in each group will have 80% power to detect a difference in means of 15.0 (Bayley's MDI), assuming that the common SD is 26.0 by using a 2-group *t*-test with a 0.05 2-sided significance level. The value for the SD was taken from a previous study (11) that reported an 18-point difference in MDI between the thyroxine and placebo groups at 2 years.

### Recruitment

Parents were aware of the possibility of a follow-up study when they were recruited to the initial TIPIT from five tertiary level neonatal units (14). Mother and child were traced through the Office of National Statistics, and investigators were informed of the mother's or child's death since discharge from hospital. An investigator made an initial approach by letter when the child was approaching 3 years of age to outline the study, discuss with parents the information sheet given, and answer any immediate questions. The parents were given a period of reflection up to a month. After this, parents were asked for consent to the inclusion of their child in the project. After recruitment, a date and time were set to assess the child's developmental milestones either in a clinic setting or in the home environment. One blinded assessor evaluated neurodevelopmental outcome at 42 months in these infants by using Bayley's III Mental and Psychomotor Developmental Indices. The Bayley's III Mental and Psychomotor Developmental Indices are used to assess the developmental status of children between 1 and 42 months of age. This assessment takes 30 to 90 minutes to administer. Parents and caregivers were blinded to the allocation in the TIPIT trial. Cognitive scales on Bayley-III assess abilities such as sensorimotor development, exploration, concept, memory, and simple problem solving while the motor scale consists of fine motor and gross motor subtests. The cognitive outcome is reported as the composite cognitive score, which has a mean of 100 and an SD of 15. Scaled scores are used for the Fine and Gross Motor scores separately, which have a mean of 10 and range from 1 to 19; this method of assessment has been validated and shown to provide reliability at this age in the prediction of final neurodevelopment (18,19).

### Analyses

Outcomes were measured as continuous variables. The difference between the 2 intervention groups was compared by using a 2-group *t*-test, and the difference in means presented with a 95% confidence interval. Assumptions of normality were checked by using a visual inspection of a histogram. Correlations were analyzed by using Spearman's correlation. The relationship between plasma fT4 concentrations and outcomes was explored by using an approach described by van Wassenaer *et al.* (20). Thus, the mean values of fT4 measurements at baseline, days 14, 21, and 28 were calculated and the effect of very low plasma fT4 concentrations was determined by measurements taken from babies in the placebo group; measurements from infants who had plasma fT4 concentrations in the lowest quartile were compared with those who had plasma fT4 concentrations in the upper 3 quartiles by using a 2-group *t*-test. The reasons were to explore the possible effects of the very low plasma fT4 concentrations on outcomes. A *p*-value of 0.05 or less was used to declare statistical significance for all analyses without adjustment for multiple comparisons.

### Ethics approval

The study was approved by North West Research Ethics Committee (reference no. 07/MRE08/37) and by the Medicines for Human Regulatory Agency (MHRA).

## Results

**FIG. 1.**
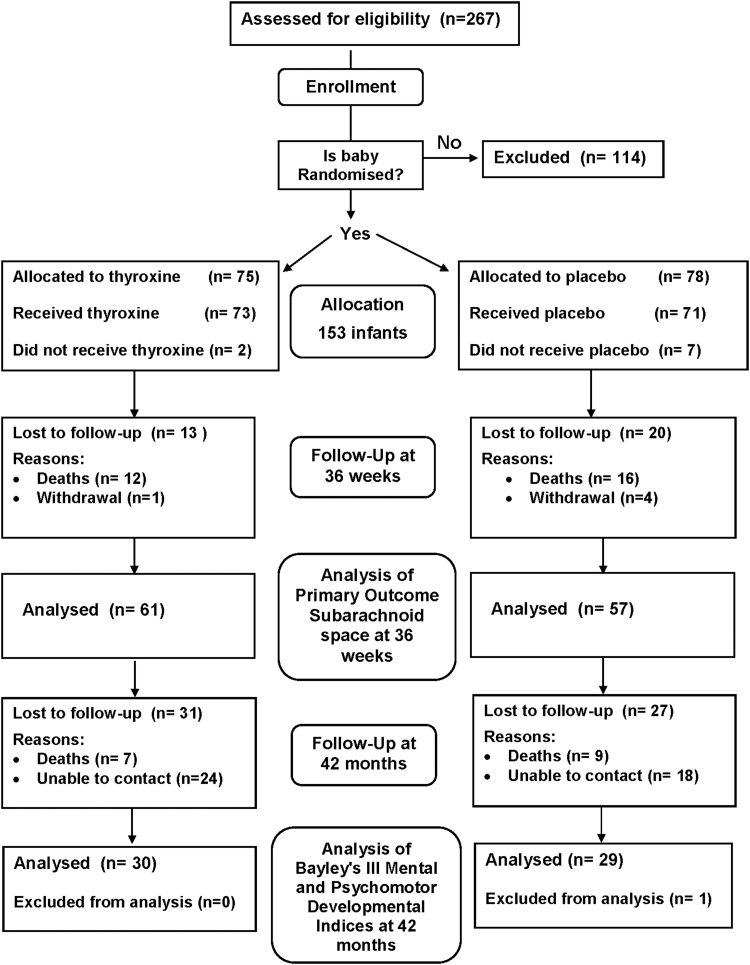
Consort diagram.

Two hundred sixty-seven participants in TIPIT from 5 participating centers were assessed for eligibility, and 153 infants were recruited to this follow-up study (14). Among infants recruited to the follow-up study, 78 infants received LT4 supplementation and 75 received placebo. There were no differences in baseline characteristics between eligible infants recruited to the follow-up study and infants not recruited to the follow-up study. The CONSORT flow diagram (Fig. 1) shows the passage of all recruited and eligible participants. There were 16 deaths (10.4%) between discharge from hospital and the approach to families about follow-up, and 42 patients were unable to be contacted despite our best efforts. One patient had to be excluded due to a mis-labeled assessment. There was a large loss to follow-up despite every effort made to contact the families via their national health insurance numbers and general practitioner practices. However, there were no significant differences in demographics between the two groups on analysis.

Baseline demographic characteristics between LT4-supplemented and placebo groups in the follow-up study are shown in Table 1, and there were no significant differences between the treatment and placebo groups. There were no differences in baseline characteristics or demographics between eligible infants recruited from the five participating centers and no differences between groups in number of complications.

**Table 1.** Demographics and Baseline Characteristics

	LT4-supplemented (*n* = 30)	Placebo (*n* = 29)
Infants less than 28 weeks' gestation randomized	75	78
Sex (males)	44 (59%)	45 (58%)
Birth weight (g)	820.7 (183.9)	842.2 (200.0)
Gestational age (weeks)	25.8 (1.3)	25.8 (1.4)
CRIB score	5.7 (3.5)	5.4 (4.1)
No. of twin births (pairs)	4 (5.3%)	6 (7.7%)
Infant baseline fT4 (pmol/L)	12.2 (6.9)	10.5 (6.3)
Infant baseline leg length (mm)	38.7 (7.1)	39.54 (6.8)
Maternal age (years)	28.8 (5.8)	28.9 (5.8)
Died	7	9
Unable to contact	24	18
Mis-labeled assessment	0	1
Cohort assessed by Bayley's III mental and psychomotor developmental indices at 42 months	30	29
Cohort assessed by subarachnoid space width at 36 weeks	61	57

Data are expressed as mean (SD) for continuous outcomes and *n* (%) for dichotomous variables.

CRIB, clinical risk index for babies; fT4, free thyroxine; LT4, levothyroxine; SD, standard deviation.

Neurodevelopmental outcomes at 42 months were evaluated by using Bayley's III Mental and Psychomotor Developmental Indices, which found that infants in the treated group performed significantly better in all 3 domains for motor, language, and cognitive function (Tables 2 and 3). Correlations between neurodevelopmental outcomes and subarachnoid space width at 36 weeks showed significance only in the motor domain (Table 4). No significant differences were noted between neurodevelopmental outcome and auxological measures such as head circumference or height and weight. MRI at term equivalence correlated with cognitive and motor composite outcomes at 42 months (Tables 5 and 6). Correlations between mean fT4 plasma concentrations in the first 4 weeks of age and neurodevelopmental outcomes at 42 months of age are shown in Table 7.

**Table 2.** Bayley's III Composite Scores at 42 Months of Age of All Infants

N = 59	Mean ± SD	Median (range)
Age (months)	42 ± 1.0	42 (40–43)
Cognitive	88 ± 12.2	90 (85–110)
Language	88 ± 17.3	94 (47–112)
Motor	81 ± 12.7	82 (73–107)

**Table 3.** Bayley's III Composite Scores at 42 Months of Age Between Treated Group and Placebo Group

	LT4-supplemented (*n* = 30)	Placebo (*n* = 29)	Mean diff [CI]	p
Age (mean ± SD)	42.1 ± 0.8	41.7 ± 1.3	0.4 [−1.8 to 2.1]	0.482
Head circumference (cm)	51.5 ± 8.7	50.2 ± 2.3	1.35 [−2.2 to 4.9]	0.443
Height (cm)	96.2 ± 8.3	98.0 ± 7.5	−1.8 [−6.2 to 2.7]	0.426
Weight (kg)	14.0 ± 4.0	15.7 ± 3.1	−1.7 [−3.8 to 0.4]	0.110
Motor	84 ± 12	77 ± 13	6.96 [0.55 to 13.38]	**0.034**
Language	92 ± 13	83 ± 20	8.93 [0.16 to 17.70]	**0.041**
Cognitive	91 ± 10	85 ± 13	6.35 [0.14 to 12.55]	**0.045**

Bold values indicate *p* < 0.05.

CI, 95% confidence interval.

**Table 4.** Correlations Between Subarachnoid Space Width at 36 Weeks and Bayley's III Composite Scores for Neurodevelopmental Outcome at 42 Months

	R^2^	p	N
Motor	−0.28	**0.032**	59
Language	−0.19	0.161	59
Cognitive	−0.09	0.481	59

Bold values indicate *p* < 0.05.

**Table 5.** Magnetic Resonance Imaging at Term Equivalence Correlates with Bayley's III Motor Composite Outcome at 42 Months

		R^2^	p	N
Internal capsule-right	FA	0.45	**0.012**	39
Internal capsule-right	Fiber number	0.34	0.070	39
Internal capsule-right	Mean fiber length	0.37	**0.043**	39
Internal capsule-left	Mean fiber length	0.36	**0.052**	30

Bold values indicate *p* < 0.05.

FA, fractional anisotropy.

**Table 6.** Magnetic Resonance Imaging at Term Equivalence Correlates with Bayley's III Cognitive Composite Outcome at 42 Months

		R^2^	p	N
OLR	ADC	−0.33	0.07	39
CCP	FA	0.44	**0.027**	39
FLR	FA	0.37	**0.045**	39

Bold values indicate *p* < 0.05.

ADC, apparent diffusion coefficient; CCP, corpus callosum posterior; FLR, frontal lobe right; OLR, occipital lobe right.

**Table 7.** Correlations Between Mean Plasma fT4 Concentrations in the First 4 Weeks After Birth and Bayley's III Composite Scores for Neurodevelopmental Outcome at 42 Months

	N	R^2^	p
Motor	48	0.25	0.084
Language	48	0.27	0.069
Cognition	48	0.35	0.016

## Discussion

This extension to the TIPIT trial was designed to explore the effects and mechanisms of thyroxine supplementation on neurodevelopmental outcomes. Extremely preterm infants in the supplemented group performed significantly better in all 3 domains of the Bayley's III Mental and Psychomotor Developmental Indices at 42 months. Overall, the Bayley's mean and median scores were noted to be lower compared with other preterm infant studies; however, direct comparisons are difficult to make as our population was more premature compared with some studies that included less premature infants (21), whereas in other studies neurodevelopmental assessment was undertaken at a younger age (22).

Surrogate outcomes are important elements in the evaluation of interventions. Given the complexity of long-term outcome studies, TIPIT was designed by using a single surrogate outcome: depth of subarachnoid space. This was selected because it had been validated and was associated with differences between groups defined by gestational age (23). The results of this study lend partial support to this choice: Subarachnoid space width measured at 36 weeks was associated with motor outcomes at 42 weeks. Low ADC and high FA values were associated with more extensive myelination. Other studies have reported on the potential of DTI to identify antecedents of disordered brain microstructure among neonates born at extreme prematurity (24). Rose *et al.* found that abnormal neurodevelopment at 18 months' corrected age was associated with lower FA values in the right posterior limb of the internal capsule (25). Van Kooij *et al.* have also reported that higher FA in the corpus callosum was associated with better cognitive performance at two years of corrected age (26). Our study shows that FA at term correlated significantly with motor and cognitive composite scores at 42 months of age. This is a unique study reporting associations between thyroid hormone status, long-term neurodevelopmental outcomes, and DTI MRI variables in infants born at extreme prematurity. These data support the use of subarachnoid space and DTI measures in future studies.

The assessment of exposure to thyroxine supplementation and outcomes showed a relationship between higher plasma fT4 concentrations during the weeks after birth and better neurodevelopmental outcomes at 42 months. These differences, however, only reached statistical significance for cognition composite scores and not for motor and language composite scores in the Bayley's III Mental and Psychomotor Developmental Indices. The assessment of exposure was relatively crude but is consistent with our other data. Of note, an MRI nested study of TIPIT found that infants with higher fT4 levels had lower ADC measurements at term equivalence, suggesting greater organization of the white matter consistent with increased myelination (15). The relationship between early plasma fT4 concentrations and outcomes on brain MRI measurements at term equivalence and at development of 42 months suggests that a future study should target infants with low plasma concentrations of fT4.

Our findings are consistent with several observational studies that have documented associations between low thyroid hormone levels in the first weeks of life and abnormal neurodevelopmental outcomes (27–29). Reuss *et al.* reported a 4.4-fold increase in the risk of disabling cerebral palsy at 2 years of age associated with low thyroid hormone levels in the early weeks of life of preterm infants born before 29 weeks' gestation (30). Another study reported that early replacement therapy in 58 low-birth-weight newborns can be beneficial for quicker motor development and better mental development in the 7th year of life (13). A Cochrane review of observational studies did not support the use of prophylactic thyroid hormones in preterm infants to reduce neonatal mortality, neonatal morbidity or improve neurodevelopmental outcomes and concluded that an adequately powered clinical trial of thyroid hormone supplementation in very preterm infants was required (31). However, early thyroxine treatment of children with Down's syndrome did not seem to benefit mental or motor development later in life in another randomized controlled trail. However, the positive effect on growth was measurable, especially in infants with an elevated plasma thyrotropin concentration in the neonatal period (32). It remains unclear whether early thyroxine exposure has simply altered the tempo of development and not the ultimate outcome.

A limitation of this study is the large number of infants lost to follow-up at 42 months, which did not reach the sample size calculation despite every effort being made to contact the families. No significant differences in demographics were noted between the two groups. The original recruitment process was also consecutive; therefore, all mothers/parents were asked for consent to recruitment. Due to the nature of the study, 43% were excluded if infants did not consent or did not fulfil the exclusion criteria of the study protocol.

This study is a randomized clinical trial specifically targeting extremely premature infants with thyroxine supplementation, and it is the third largest randomized trial of thyroid hormone supplemented to infants born below 28 weeks' gestation. MRI DTI measurements in infants with higher fT4 plasma concentrations during early life had lower ADC measurements at term equivalence, suggesting greater organization of the white matter. Future larger scale trials would seem to be justified on the basis of the results reported here; these could usefully target preterm infants on the basis of their initial thyroid hormone levels. This study suggests that early supplementation with LT4 may improve long-term neurodevelopment in infants born below 28 weeks' gestation, but larger trials are needed and the modest improvements shown are not strong enough to warrant a change in practice.

## Supplementary Material

Supplemental data
